# High-resolution 3D X-ray diffraction microscopy: 3D mapping of deformed metal microstructures

**DOI:** 10.1107/S1600576722007361

**Published:** 2022-08-30

**Authors:** Mustafacan Kutsal, Henning Friis Poulsen, Grethe Winther, Henning Osholm Sørensen, Carsten Detlefs

**Affiliations:** a Department of Physics, Technical University of Denmark, 2800 Kongens Lyngby, Denmark; b The European Synchrotron, 38043 Grenoble, France; c Department of Civil and Mechanical Engineering, Technical University of Denmark, 2800 Kongens Lyngby, Denmark; d Xnovo Technology ApS, 4600 Køge, Denmark; Ecole National Supérieure des Mines, Saint-Etienne, France

**Keywords:** X-ray imaging, X-ray diffraction, 3DXRD, structural materials

## Abstract

Three-dimensional X-ray diffraction microscopy, 3DXRD, has become an established tool for orientation and strain mapping of bulk polycrystals. However, it is limited to a finite spatial resolution of ∼1.5–3 µm. Presented here is a high-resolution modality of the technique, HR-3DXRD, for 3D mapping of submicrometre-sized crystallites or subgrains with high spatial and angular resolution. Specifically, the method is targeted to visualization of metal microstructures at industrially relevant degrees of plastic deformation. Exploiting intrinsic crystallographic properties of such microstructures, the high resolution is obtained by placing a high-resolution imaging detector in between the near-field and far-field regimes. This configuration enables 3D mapping of deformation microstructure by determining the centre of mass and volume of the subgrains and generating maps by tessellation. The setup is presented, together with a data analysis approach. Full-scale simulations are used to determine limitations and to demonstrate HR-3DXRD on realistic phantoms. Misalignments in the setup are shown to cause negligible shifts in the position and orientation of the subgrains. Decreasing the signal-to-noise ratio is observed to lead primarily to a loss in the number of determined diffraction spots. Simulations of an α-Fe sample deformed to a strain of ε_*vM*_ = 0.3 and comprising 828 subgrains show that, despite the high degree of local texture, 772 of the subgrains are retrieved with a spatial accuracy of 0.1 µm and an orientation accuracy of 0.0005°.

## Abstract 2

A full-field X-ray diffraction contrast method is presented (high-resolution 3D X-ray diffraction), for 3D mapping of plastically deformed microstructures. The essence of the method is the introduction of a 2D detector in the optical mid-field regime. The properties and limitations of the method are estimated by numerical simulations.

## 1. Introduction

Most crystalline materials, whether they are metals, ceramics, rocks or bones, have a hierarchically organized structure with grains comprising domains which in turn contain atomic-scale defects. The agglomerate is often highly heterogeneous, with the grains and domains varying in size, shape, crystallographic orientation and stress state, as well as in their relationships to the neighbouring structure. To understand the mechanisms governing structural evolution during processing or application, it is critical to establish methods that are capable of acquiring 3D movies of bulk specimens. As millimetre-sized specimens may comprise billions of crystalline elements, a complete mapping of such specimens is impossible. Instead, a prime ambition is to be able to ‘zoom in and out’ and probe different length scales within one setting.

Currently, X-ray diffraction based on synchrotron or free-electron laser sources is the only non-destructive probe that, in general, can provide the required combination of brilliance, penetration power and sensitivity to the local crystallography. On the coarse scale, for 3D mapping of polycrystals with 5–500 µm-sized grains, tomographic approaches such as 3D X-ray diffraction (3DXRD, also called high-energy diffraction microscopy) (Poulsen *et al.*, 2001 ▸; Oddershede *et al.*, 2010 ▸; Bernier *et al.*, 2011 ▸; Hefferan *et al.*, 2012 ▸; Poulsen, 2012 ▸; Schmidt, 2014 ▸; Li & Suter, 2013 ▸; Pokharel *et al.*, 2015 ▸; Winther *et al.*, 2017 ▸; Juul *et al.*, 2017 ▸, 2020 ▸) and diffraction contrast tomography (DCT) (King *et al.*, 2008 ▸; Ludwig *et al.*, 2009 ▸; Vigano *et al.*, 2014 ▸; Zhang *et al.*, 2018 ▸) have been established. Overlap of diffraction spots on the detector limits these methods to a few thousand grains (Sørensen *et al.*, 2012 ▸), and the employed detection principles limit the spatial resolution to about 1.5–3 µm (Viganò *et al.*, 2016 ▸; Reischig & Ludwig, 2019 ▸; Bhattacharyya *et al.*, 2020 ▸).

For plastically deformed metals, this implies that the individual subgrains or cells forming inside the parent grains cannot be visualized directly. As an example, for 99.99% pure aluminium, the boundary spacing decreases continuously during cold rolling from 2 µm at a strain of ε_*vM*_ = 0.1 to below 1 µm at a strain of ε_*vM*_ = 0.8 (Hansen, 2001 ▸). Generally speaking, to characterize the deformed microstructure of metals in terms of *e.g.* misorientation angles, a spatial resolution of the order of 300 nm is required for industrially relevant materials.

In attempts to overcome these limitations novel 3DXRD modalities and improved data analysis procedures have been presented (Hayashi *et al.*, 2015 ▸; Hektor *et al.*, 2019 ▸; Nygren *et al.*, 2019 ▸, 2020 ▸; Reischig & Ludwig, 2019 ▸). In particular, a scanning 3DXRD method is realized by collecting far-field 3DXRD images while raster scanning the sample with a pencil beam. Then, 3D maps of intragranular strain fields are reconstructed using tomographic approaches (Hayashi *et al.*, 2015 ▸; Henningsson *et al.*, 2020 ▸) with a voxel size of ∼0.5 µm. However, the scanning approach inherently makes this modality slow.

Limitations for observing deformation microstructures can partly be overcome by dark-field X-ray microscopy (DFXM), as introduced by Simons *et al.* (2015 ▸). By placing an X-ray objective in the Bragg diffracted beam of a crystalline element of choice, a magnified real-space image is created on a high-resolution 2D detector. DFXM can map the local orientation and axial strain of domains in three dimensions (Simons *et al.*, 2016 ▸, 2018 ▸; Ahl *et al.*, 2017 ▸; Mavrikakis *et al.*, 2019 ▸) with a resolution of ∼100 nm using a compound refractive lens as objective. This has allowed mapping of embedded subgrains in metals deformed as high as ε_*vM*_ = 2 (Yildirim *et al.*, 2022 ▸). However, the part of a pole figure that can be covered within a time frame of an hour is of the order of 10 × 10°. Thus, applying DFXM to medium to heavily deformed metals gives rise to maps of the microstructure that are incomplete.

As an alternative to the 3DXRD and DFXM methods described above, scanning Laue (Larson *et al.*, 2002 ▸; Hofmann *et al.*, 2013 ▸; Schroer *et al.*, 2005 ▸; Mimura *et al.*, 2009 ▸; Ice *et al.*, 2011 ▸; Xu *et al.*, 2017 ▸) and coherent (Miao *et al.*, 1999 ▸; Dierolf *et al.*, 2010 ▸; Shapiro *et al.*, 2005 ▸; Chapman & Nugent, 2010 ▸; Miao *et al.*, 2015 ▸; Chamard *et al.*, 2010 ▸; Yau *et al.*, 2017 ▸) X-ray methods are rapidly progressing towards 10 nm resolution. Overlap of diffraction signals, however, limits these to small sampling volumes, excluding studies on multiple length scales. The acquisition of 3D movies remains a challenge.

In this article we introduce a high-spatial-resolution generalization of the 3DXRD method, HR-3DXRD, with the aim of providing comprehensive 3D mapping at a resolution of a few hundred nanometres. The method is specifically designed to visualize the microstructure of plastically deformed metals. The HR-3DXRD method relies on detector technology that is not yet mature. It is motivated by the expected realization of new 2D detectors with more than 100 megapixels. For this reason, we describe the experimental strategy and demonstrate the feasibility of the proposed analysis scheme on simulated diffraction patterns based on tessellations representing typical deformed microstructures. The concept of HR-3DXRD relies on the fact that, during the deformation process, grains organize into arrays of subgrains separated by low-angle boundaries, whereas the bulk of the subgrains remain relatively free of dislocations and thus maintain a high crystallographic perfection.

HR-3DXRD determines the centre of mass (CoM) position, the volume and the orientation of all subgrains within a given volume within the specimen. Volumetric mapping is then provided by tessellation, a procedure that is known to provide very accurate representations of deformed microstructures (Lyckegaard *et al.*, 2011 ▸; Alpers *et al.*, 2015 ▸; Quey & Renversade, 2018 ▸).

We first present the perceived experimental configuration for HR-3DXRD and the foreseen reconstruction route. We compare this new modality with existing 3DXRD modalities. We then demonstrate how a data analysis workflow for HR-3DXRD can be established using existing 3DXRD software, even though the HR data fall outside of the intended range of applicability of these. Next, the quality of the 3D mapping and the limitations of the method are explored using full-scale simulations. Finally, we discuss certain aspects of an experimental implementation to comment on feasibility. The results of the first experiments have been reported elsewhere (Kutsal, 2021 ▸).

## 2. The HR-3DXRD method

### 2.1. High-resolution methodology

**Figure 1:**
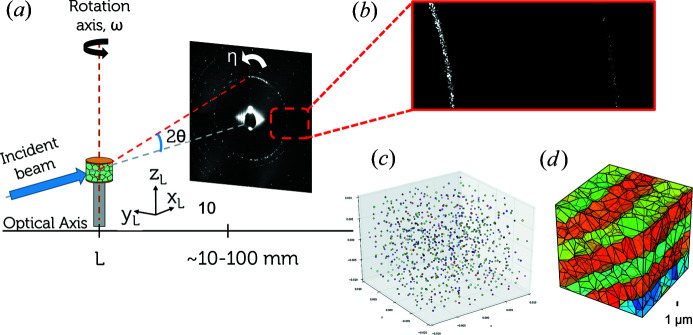
(*a*) An illustration of the conceptual experimental setup of HR-3DXRD. (*b*) An example of simulated diffraction data within a region of interest. (*c*) A reconstructed 3D map of subgrains, each associated with an orientation, a CoM and a volume (encoded in the colour) and (*d*) a volumetric map provided by subsequent tessellation (here the colours symbolize orientation).

The underlying principle of HR-3DXRD is illustrated in Fig. 1 ▸. As for far-field 3DXRD, a monochromatic and parallel X-ray beam from a synchrotron source impinges on a sample. Let the wavelength be λ. Images are acquired while rotating the sample around an axis (ω) perpendicular to the incoming beam. The diffracted signal from the illuminated (sub)grains is detected on a 2D detector. The key difference from far-field 3DXRD, however, is that for HR-3DXRD this detector has a pixel size of about 3 µm (instead of 50–200 µm) and is located at a distance *L* in between the near and far field (instead of the far field, *L* > 500 mm). Specifically, the sample-to-detector distance *L* is set so as to fulfil the criterion that the Fresnel number 

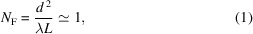

where *d* is the average subgrain size. As discussed below, this condition ensures that data are acquired with an optimal compromise between spatial and angular resolution.

For a given ω setting all subgrains fulfilling the Laue condition will give rise to diffraction spots on the detector [Fig. 1 ▸(*b*)]. For each isolated diffraction spot, the CoM position on the detector is determined. This can be done with an accuracy that is substantially better than the pixel size. At the same time, the integrated intensity of the spot is determined.

During the entire ω scan, each subgrain will generate multiple diffraction spots, and in total hundreds of thousands of spots may be registered. On the basis of their CoM detector coordinates and associated ω values, the HR-3DXRD software provides a multigrain indexing: the spots are sorted according to their subgrain of origin, while at the same time determining the CoM position of each subgrain, its average orientation and (optionally) a strain tensor. The resulting 3D map is illustrated in Fig. 1 ▸(*c*). Within the 3DXRD nomenclature introduced by Poulsen (2020 ▸), this is a Mode II map.

Finally, to produce a site-filling map the volume is tessellated. This has been done successfully in the past on the grain scale using Mode II 3DXRD data. An example of such a 3D tessellated map is shown in Fig. 1 ▸(*d*). Specifically, Lyckegaard *et al.* (2011 ▸) demonstrated that Laguerre tessellations provide a good resemblance to actual microstructures. A refined approach has recently been demonstrated to account correctly for about 90% of the voxels in undeformed polycrystals investigated by DCT (Quey & Renversade, 2018 ▸). For the future, Alpers *et al.* (2015 ▸) presented a general linear programming model for computing globally optimal tessellations. It is clear that the planar boundaries in the initial phantom cannot be reproduced by a tessellation. Nevertheless, due to the systematically alternating misorientations typical of deformed microstructures the main features are still captured.

### 2.2. Comparison with existing 3DXRD modalities

**Figure 2:**
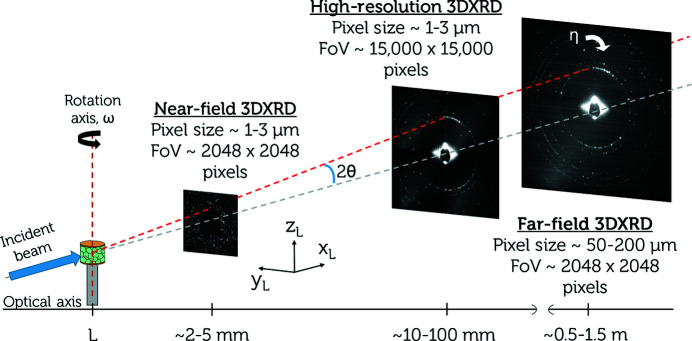
A comparison of the setup of HR-3DXRD with those of existing near-field 3DXRD and far-field 3DXRD methods. The sample-to-detector distance *L*, the field of view and the pixel size for the three modalities are shown. The angles (2θ, η, ω) are defined, as is the laboratory coordinate system (*x*_L_, *y*_L_, *z*_L_).

The experimental configuration for HR-3DXRD is compared in Fig. 2 ▸ with existing setups known as near-field 3DXRD and far-field 3DXRD. As mentioned above, the main novelty is the number of pixels on the detector.

To compare the different regimes, assume that the beam diffracted by a given (sub)grain of size *d* has a divergence ξ. The spot size Δ*s* at a distance *L* is then given by Δ*s* ^2^ = *d* ^2^ + *L* ^2^ξ^2^. The spot position on the detector is *s* = *x* + *L*θ, where *x* is the linear position of the grain and θ the scattering angle of the diffracted beam relative to the optical axis. Experimentally, a diffraction spot’s CoM position can be determined to within a fraction ε of the spot size, say ε = 5%. The corresponding uncertainties in the linear position *x* and scattering angle θ are then 

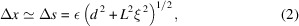




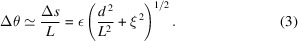

The linear position can be determined most precisely in the near field, 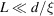 , whereas the scattering angle can be determined most precisely in the far field, 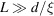 . Acceptable *simultaneous* accuracy in both linear position and scattering angle requires a compromise, *d*/(*L*ξ) ≃ 1. In the ideal diffraction-limited case the diffracted beam’s divergence is given by ξ ≃ λ/*d*, such that *d*/(*L*ξ) ≃ *d* ^2^/(*L*λ) = *N*_F_, which is the Fresnel number. The Fresnel number can thus be used to distinguish the regimes, even if coherent diffraction effects are not explicitly considered. Note that coherent or wave-optical effects that occur in the near field do not affect the CoM position of a diffraction spot, which always propagates in a straight line (Ehrenfest, 1927 ▸; Paganin, 2020 ▸).

Characteristic of the far-field setup is that the position of diffraction spots on the detector is dominated by the angle of the diffracted beam (governed by grain orientation and strain state), with the point of origin (real-space CoM position of the grain) as a small correction. In this case, ‘diffraction’ detectors are used, with pixel sizes in the range 50–200 µm and sample-to-detector distances *L* correspondingly in the range 500–1500 mm. While this provides high efficiency and high resolution of orientations and strain tensors as averaged over each grain, the spatial resolution is limited to about 10 µm in this mode. The Fresnel number 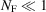 , corresponding to the Fraunhofer regime.

In the near-field limit, the position of the ‘spot’ on the detector is dominated by the real-space position and shape of the grain. The angular variation of the diffracted beam from a grain (orientation gradients and strain state) is a small correction that mostly leads to blurring of the spots. Typically, a high-resolution 2D detector with pixel sizes of about 1 µm is used and *L* ≃ 10 mm. In this mode the achievable spatial resolution is improved to around 1.5–3 µm (Ludwig *et al.*, 2009 ▸; Sun *et al.*, 2018 ▸; Bernier *et al.*, 2020 ▸; Zhang *et al.*, 2020 ▸) while the angular resolution is degraded to the point that strain measurements are compromised. In this case the Fresnel number *N*_F_ ≫ 1. In principle the effective resolution may be increased by mapping only the CoM positions and volumes – *i.e.* Mode II operation – but in practice the diffraction spots emerging from the large number of simultaneously illuminated subgrains cannot be separated and the diffraction pattern becomes a set of continuous Debye–Scherrer rings, typical of classical powder diffraction. In this limit the multigrain analysis approach underlying 3DXRD cannot be pursued.

In HR-3DXRD the detector is placed midfield, with *N*_F_ ≃ 1, where a good compromise between linear and angular accuracy is obtained. For an expected subgrain size *d* ≃ 1 µm and a wavelength λ = 1 Å, *N*_F_ = 1 yields a sample-to-detector distance *L* ≃ 10 mm. Under ideal conditions, the spot size at this distance is approximately twice the subgrain size. To avoid spot overlap, the detector pixels should be of a similar size. Therefore one of the main challenges for the practical implementation of HR-3DXRD is to find a suitable detector. With a pixel size of 3 µm and a typical setup (detailed below in Section 3) the number of pixels required is 15 000 times 15 000, as illustrated in Fig. 2 ▸. The hardware implementation of this approach is beyond the scope of this article, but some options will be discussed in Section 5.

### 2.3. Mapping the microstructure of plastically deformed metals

The microstructure of plastically deformed metals has several distinctive features of importance to the HR-3DXRD approach. We start by summarizing these pertinent features.

**Figure 3:**
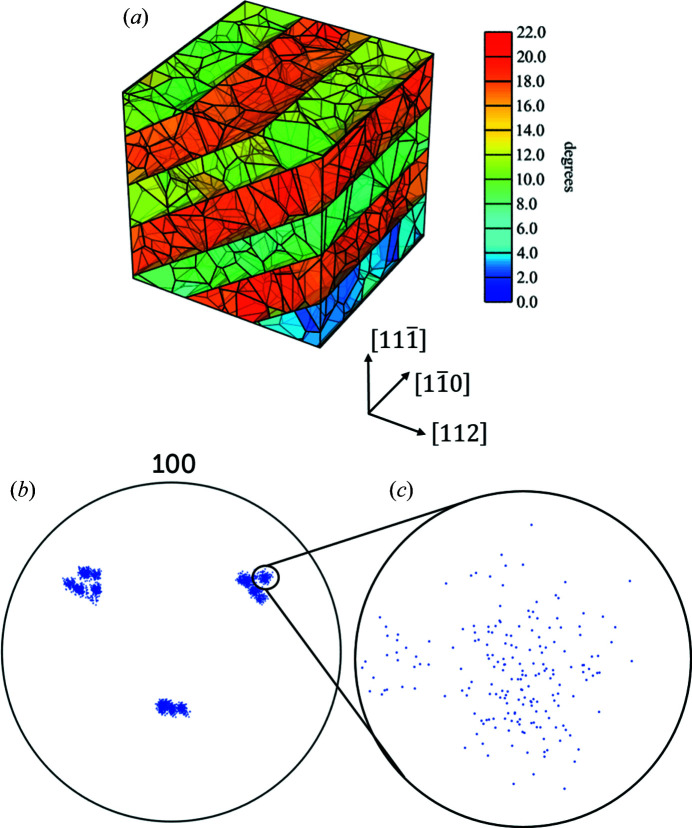
(*a*) A phantom representing the hierarchical structure of deformed metals. Subgrains are contained in a cube-shaped box with a side length of 20 µm. Colours mark the misorientation relative to a reference orientation in the outskirts of the orientation spread. The phantom exhibits 828 subgrains delineated by six GNBs aligned with the (001) plane. (*b*) The corresponding {100} pole figure. (*c*) A close-up of the {100} pole figure. The diameter of the circle is ∼10°. The narrow misorientation between subgrains leads to a high density of reflections around the average orientation.

At deformation degrees above ε_*vM*_ = 0.05, metals with a medium to high stacking-fault energy present a hierarchical microstructure, where the grains are subdivided by planar boundaries known as geometrically necessary boundaries, GNBs. Subgrains (with boundaries known as incidental dislocation boundaries, IDBs) are found in between the GNBs. The orientation difference across GNBs is larger than that across the IDBs, as illustrated by the colours in Fig. 3 ▸ where the subgrains between GNBs form bands. This phantom was used in the simulations discussed in this paper. Further details are given in Section 4.2.

In relation to HR-3DXRD, we note the following:

(i) *Subgrains are near-perfect crystals.* The implication is that the intrinsic angular spread is close to being governed by the diffraction limit. Hence, spot overlap can be minimized by increasing the sample-to-detector distance until *N*_F_ = 1, the fundamental insight underlying HR-3DXRD. This fact was experimentally demonstrated recently in a diffraction experiment on the recovery of Al by Ahl *et al.* (2020 ▸).

(ii) *CoM positions are highly accurate*. The subgrains have regular shapes, implying that all diffraction spots tend to be approximately Gaussian. Provided that the count rates are sufficient, it is well known in crystallography that the CoM position of such spots on the detector can be found with an accuracy of 0.1 times the size of the spot or better. Moreover, the position of each subgrain is derived from a multitude *N* of diffraction spots, and the resulting accuracy of the position of the subgrain is expected to vary as 1/*N* ^1/2^.

(iii) *The diffraction pattern is multiscale*. The largest units (the grains), the medium-scale units (the bands) and the smallest-scale units (the subgrains) exhibit misorientations of tens of degrees, a few degrees and ∼1°, respectively. This implies that the diffraction signals from a single grain of choice can be separated from those from other grains. Moreover, in favourable conditions the diffraction from individual bands may also be separated. These separations simplify the data analysis chain. On the downside, the sharp texture within a band strongly increases the probability of spot overlap, as illustrated by Fig. 3 ▸. This is foreseen to be the main limitation of HR-3DXRD, provided suitable detectors become available.

## 3. Data analysis

**Figure 4:**
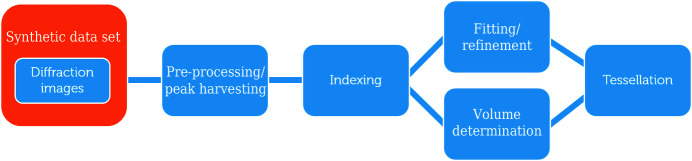
A flow diagram of the HR-3DXRD data analysis pipeline.

The generic data analysis pipeline foreseen for HR-3DXRD is similar to that for grain mapping based on Mode II far-field 3DXRD data [see *e.g.* Oddershede *et al.* (2010 ▸)], as illustrated in Fig. 4 ▸, with subgrains replacing grains as the basic crystallographic entities. However, the existing algorithms for indexing were not established with HR-3DXRD in mind; the angular specifications in HR-3DXRD are an order of magnitude more stringent than in classical 3DXRD and existing software has never been applied to the very high degrees of texture exhibited locally within bands (*cf*. Fig. 3 ▸). As such, we foresee that new indexing algorithms may be required.

In this article, we pursue the use of existing far-field 3DXRD software. In the following, we establish and verify a data analysis pipeline along the lines of Fig. 4 ▸ by means of geometric optics simulations.

Specifically, synthetic data are generated with the *PolyXSim* software of the *FABLE* package (Sørensen, 2008 ▸) based on a phantom describing the microstructure in terms of the CoM position, orientation and volume of the subgrains. Given a specified experimental setup, *PolyXSim* simulates scattering vectors and uses ray tracing of the subgrain CoM positions to determine the CoM detector positions of the associated diffraction spots.^1^ The exact shape of the diffraction spot cannot be simulated but a (bounding) area can be specified, representing the combined effect of all sample and instrumental contributions to the broadening. In this way, spot overlap is modelled.

We shall assume that other grains have an orientation such that the diffraction signals do not overlap and we will, therefore, focus on a single deformed grain.

In the following, we describe the steps of data analysis flow and its validation.

### 3.1. Material of choice

The material of choice is pure α-Fe with a unit-cell parameter of 2.856 Å (space group No. 229, 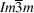 ). The structure of each subgrain is assumed to be perfect with zero strain. Several phantoms are used with a different number of subgrains, as described below.

### 3.2. Synthetic data generation

The experimental geometry was chosen to represent a physical experiment as much as possible. Synthetic data were generated from a representative subgrain ensemble having specified CoM positions, orientations and corresponding volumes. An ideal parallel and monochromatic incident beam was assumed at an X-ray energy of 52 keV (λ = 0.23843 Å). A distortion-free 2D detector with field of view (FoV) of 15 000 × 15 000 pixels and a pixel size of 2.93 µm was placed 70 mm away from the sample.^2^ For an average subgrain size of 1.3 µm this corresponds to *N*_F_ = 1. The centre of the FoV was aligned with the optical axis and the three possible detector rotations were set to zero. ω scans were performed over the range 0–360° with a fixed step size of 0.1°.

The generated data consist of a list of normalized scattering vectors and a set of synthetic diffraction images (as .tif f files). Following the convention introduced by Poulsen (2004 ▸), the scattering vectors **G**_*l*_ are expressed as 

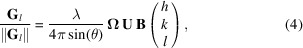

where θ is the Bragg angle, 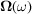 is the rotation matrix, **U** represents the orientation of the subgrains with respect to the ω rotation axis, the matrix **B** contains the reciprocal-lattice parameters, and *h*, *k* and *l* are the Miller indices. The scattering angles of the diffraction spots on the synthetic diffraction images are derived from the calculated normalized scattering vectors by considering the adopted experimental geometry. The generated diffraction spots are assumed to have a 3D Gaussian shape with an assumed ω spread of the adopted ω step size, 0.1°. The generated images have no fluctuating background and no noise. For some of the numerical studies, Poisson noise is added (see below). With the mentioned configuration, synthetic diffraction images contain six full and six partial Debye–Scherrer rings. Each pixel corresponds to 0.002° in the 2θ direction.

The file size of a single synthetic image is 450 MB, adding up to 1.6 TB for the simulation of a full rotation scan.

### 3.3. Data analysis

#### 3.3.1. Image processing and peak harvesting

The procedures for identifying diffraction spots and measuring their integrated intensities and CoM positions on the detector are similar to those of X-ray diffraction from multicrystals, in general. In order to determine the position of the reflections from these images, the *peaksearch* module of the package *FABLE* (Wright, 2005 ▸) was used. The scattering vectors were then calculated with the appropriate global parameters.

#### 3.3.2. Indexing

Indexing using existing 3DXRD indexing engines was not straightforward because these programs have not been constructed for samples with a very high degree of local texture in the deformed microstructure. However, a main result of this work is that the *Grainspotter* software (Schmidt, 2014 ▸) can actually be employed, provided that the 2θ, η and ω tolerances are set very tight. From the scattering vectors provided, *Grainspotter* identified candidate subgrains by indexing their orientation along with fitting their CoM position in 3D. For the cases presented in this study, different sets of tolerances and cuts were used. The relevant parameters will be given in their respective sections.

#### 3.3.3. Fitting and refinement

The CoM positions of the subgrains and their orientations were subjected to refinement by the *makemap* module of *ImageD11* (Wright, 2005 ▸). Here, the assigned diffraction peaks were potentially re-assigned to other indexed subgrains by successively decreasing the *hkl* tolerance, defined as 

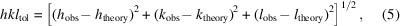

where ‘obs’ are the observed *hkl* values and ‘theory’ are the theoretically calculated *hkl* values from the parent subgrain’s orientation. The output of this refinement step consists of a list of subgrain positions and orientations, and for each subgrain a list of assigned reflections.

#### 3.3.4. Volume determination

The integrated intensities of the harvested diffraction spots are proportional to the Lorentz factor times the volume of the associated subgrain of origin. The Lorentz factor can, to a good approximation, be expressed as (Poulsen, 2004 ▸) 

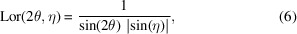

where 2θ and η are the associated scattering angles of each diffraction spot, defined in Fig. 2 ▸ and by Poulsen (2004 ▸). For each subgrain, the intensities of the associated spots are normalized to this factor. The results then undergo statistical analysis: outliers are removed and the average values determined. From this, subgrain volumes may be determined by a global normalization to the integrated intensity of the entire diffraction pattern, provided the total gauge volume is known (Lauridsen *et al.*, 2000 ▸), or by fitting the derived subgrain size distribution to results from electron backscatter diffraction (EBSD). In addition, this proportionality constant can be treated as a global fitting parameter for the tessellations.

#### 3.3.5. Volumetric mapping by tessellation

Tessellation was obtained with the Laguerre approach as implemented in *Neper* (Quey, 2019 ▸).

### 3.4. Implementation

The simulations were performed on a 16-core Intel Xeon processor E5-2680 comprising 128 GB memory. The peak harvesting for Phantom *B*, illustrated in Fig. 3 ▸, took about 16 h and the indexing and refinement steps took approximately 3 h.

## 4. Simulations

The simulations presented below aim to estimate the feasibility of HR-3DXRD by quantifying the effect of prominent error sources and providing a numerical demonstration on three phantoms with high resemblance to actual microstructures. By comparison with the original phantom the quality of the simulations is quantified by six figures of merit:

(i) The *number of subgrains indexed* compared with the number simulated.

(ii) *Purity*, the subgrain average of the ratio between the number of reflections correctly assigned to a subgrain by the indexing algorithm and the number of simulated spots for the subgrain (Schmidt, 2014 ▸).

(iii) *Completeness*, the subgrain average ratio of the number of reflections assigned (correctly or not) to a grain by the indexing algorithm and the theoretical number of simulated spots for that subgrain. (In the simulations below the completeness of subgrains turned out always to be equal to their purity. To avoid repetition, only purity values are presented here.)

(iv) The subgrain average of the *accuracy of the CoM positions* of the subgrains indexed, calculated by comparison with their ground truth counterpart.

(v) The subgrain average of the *accuracy of the orientations* of the subgrains indexed, calculated by comparison with their ground truth counterparts.

(vi) The subgrain average of the θ, η and ω errors of the assigned reflections, calculated by comparison with their counterparts in the original phantom.

To match an analysed subgrain to its counterpart in the phantom, we determine a figure of merit (FOM) for all candidate pairs comprising terms related to the calculated misorientation angle and the CoM position difference. The pair with minimum FOM is assigned as a match, provided the misorientation angle is less than 0.1° and the CoM difference is below 0.5 µm. Next, the diffraction peaks for such a pair of subgrains are matched by minimizing the distance of the corresponding diffraction vectors in reciprocal space. In the minimization process, candidate pairs of reflections are considered only if their relative distance is below 0.1 Å^−1^.

### 4.1. HR-3DXRD limitations

Similarly to classical 3DXRD, the perceived limitations of HR-3DXRD come from systematic errors in alignment, signal-to-noise issues, the limited number of reflections probed and the density of diffraction spots on the detector. The effect of geometric errors in 3DXRD was studied systematically by Menasche (2016 ▸) and Menasche *et al.* (2020 ▸). The effect of diffraction spot overlap in multigrain indexing was studied by Sørensen *et al.* (2012 ▸), showing that 10% overlap is acceptable. However, the above-mentioned studies only relate to random orientation distributions with angular tolerances that are an order of magnitude more relaxed than those of HR-3DXRD.

In the thesis work of Kutsal (2021 ▸) these issues were re-investigated in view of the high degree of local texture inherent in metal microstructures and the very high angular resolution provided by HR-3DXRD. Below we summarize the findings.

#### 4.1.1. Study of systematic errors in the geometry of the setup

In diffraction imaging experiments, the conversion of the peak positions on the detector to scattering vectors requires the geometry of the experimental setup to be known with the utmost accuracy. Hence, prior to scattering vector calculation, a set of global parameters describing the experimental configuration has to be refined. These include the sample-to-detector distance, the tilts of the detector, the beam centre position on the detector frame, the incident wavelength *etc*. In experiments, these parameters are typically determined by calibrations using suitable powders or the multigrain samples themselves. We note that the former approach provides an absolute measure for the determined crystallographic parameters, whereas the latter would provide such measurement in a relative manner, yet consistent within the analysed data set.

In a previous study, Menasche (2016 ▸) reported the effect of errors in these global parameters for a near-field high-energy diffraction microscopy experiment. By varying all global parameters in a cross-correlative manner, Menasche and co-workers found that the uncertainties in the beam centre *z* (detector *z* centre) position and detector rotation around the *z* axis (detector tilt *z*) have the strongest negative effect on the quality of the maps (Menasche, 2016 ▸; Menasche *et al.*, 2020 ▸).

**Figure 5:**
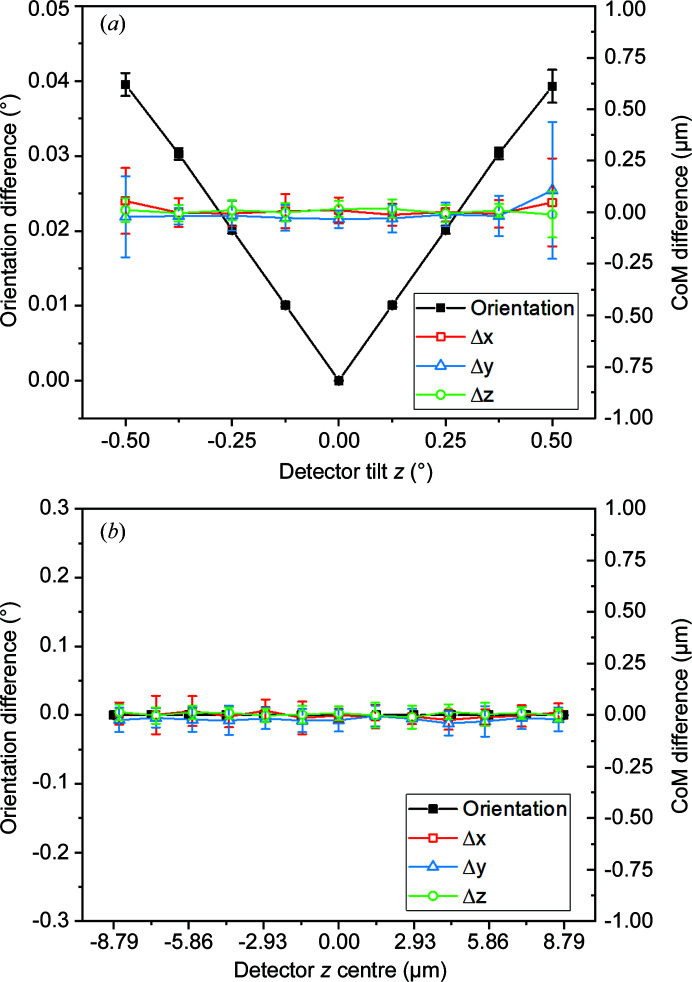
Estimation of the average errors in orientation and CoM position of subgrains as a function of misalignment of the instrument. The errors are plotted as a function of misalignment in (*a*) detector tilt *z* and (*b*) detector *z* centre. The errors represent the difference between grains in the misaligned case and matching grains for the ideal alignment. The presented CoM errors are calculated after subtracting the rigid-body translations due to the considered misalignments.

We repeat the analysis of Menasche (2016 ▸) for these two misalignment cases for our mid-field HR-3DXRD geometry. For reference, the indexing tolerances in the 2θ, η and ω angles were set to be 0.05, 0.075 and 0.05°, respectively. The rigid-body translations and rotations which have no effect on the resulting materials science were subtracted, and the resulting residual motions and orientation errors are shown in Fig. 5 ▸. The residual motions of subgrains for both detector *z* centre and detector tilt *z* mis­alignment are below 0.1 µm in all three directions, even with the relatively large ranges of misalignment investigated, implying that such residual motions can be neglected (the spatial resolution target of HR-3DXRD is 0.3 µm).

For the detector *z* centre case, the orientation errors resulting from the misalignment are below 0.001°. For the detector tilt *z* case, the orientation error and its standard deviation increase linearly with the misalignment, up to 0.04 ± 0.002° for tilt_*z*_ = ±0.5°. Note that such inaccuracies are still an order of magnitude smaller than the accuracy of conventional EBSD maps (Wilkinson & Britton, 2012 ▸; Zaefferer *et al.*, 2008 ▸; Polonsky *et al.*, 2019 ▸) and for most purposes they are negligible.

#### 4.1.2. Study of the effect of noise

The presence of noise in experimental diffraction images may lead to errors such as undetected (low-intensity) peaks, detection of false peaks (Sørensen *et al.*, 2012 ▸) and errors in peak position determination.

In order to survey the effect of noise on HR-3DXRD experiments, we adopted a noise model where the noisy synthetic image Im_noisy_ is derived from the generated ideal images Im_ideal_ as follows.

The ideal noiseless image is first scaled by an ‘intensity scale factor’ 1/β. A constant background is then added. The parameter β thus adjusts the signal-to-background ratio. As an example, increasing the exposure time of a detector with constant readout noise would correspond to decreasing β. The resulting image is scaled by a detector-specific factor 1/α, which represents the quantum efficiency with which the detector converts X-ray photons into detectable electrons. The counting statistics are simulated by running this image through a Poisson filter. The final image is obtained by re-scaling with α, allowing us to adjust the level of Poisson noise without affecting the overall intensity.

The procedure is summarized in the formula 

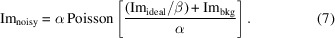

In this formulation, the constant background and the noise scaling factor are detector-specific parameters. These were estimated from experimental data. Saturation is taken into account by generation of synthetic diffraction images with a 14-bit dynamic response, similar to currently available high-resolution imaging detectors (Coan *et al.*, 2006 ▸). For details of the noise model and simulations see Kutsal (2021 ▸).

**Figure 6:**
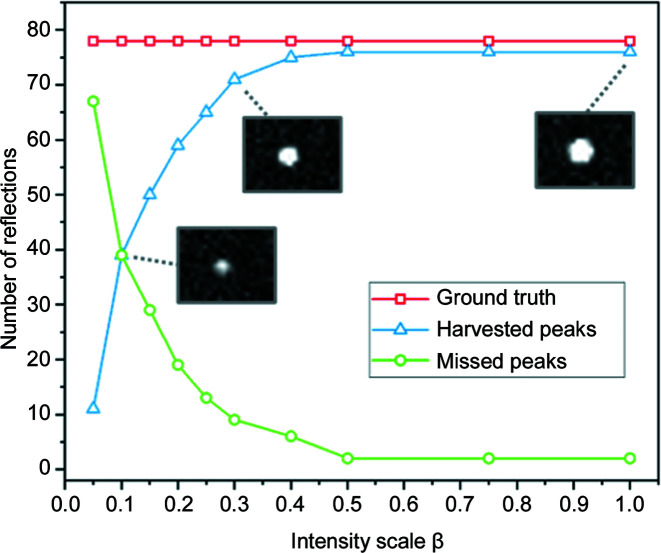
The effect of noise on the simulated diffraction peaks, I. The plot shows the number of harvested peaks with respect to the signal-to-noise ratio, expressed by the intensity scale parameter β (see text). The insets are regions of interest from corresponding images comprising the 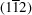 peak, to illustrate how strong the peak appears at β = 0.1, 0.3 and 1.0, with the same greyscale intensity.

The effect of the varying intensity level on the number of harvested peaks is shown in Fig. 6 ▸. Up to β = 0.4, ∼95% of diffraction peaks are successfully harvested. Further reduction of the intensity reduces the number of harvested peaks in an exponential manner.

**Figure 7:**
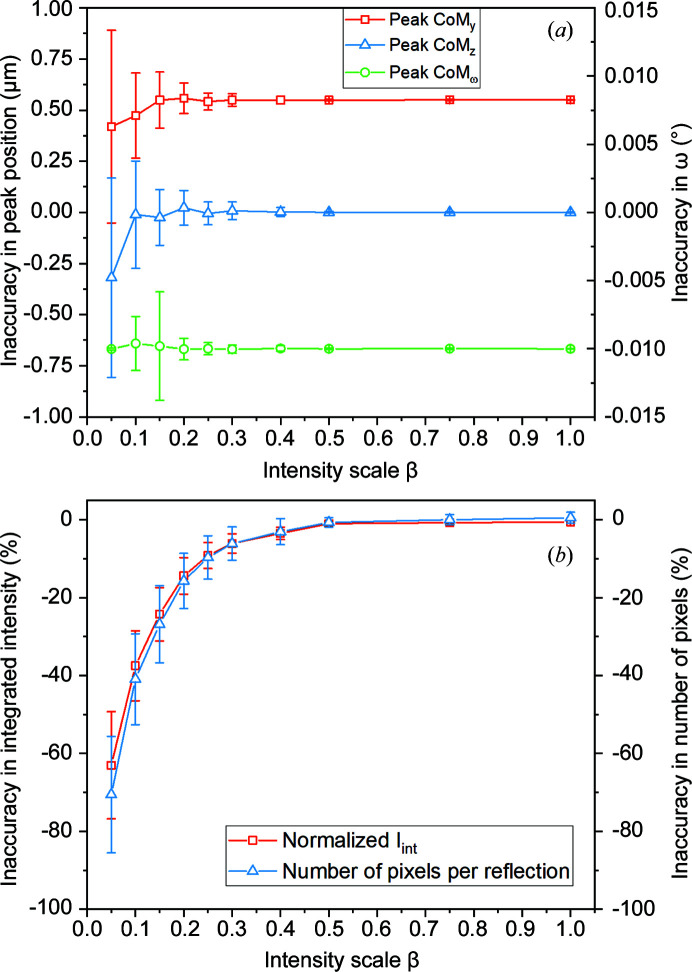
The effect of noise on the simulated diffraction peaks, II. (*a*) The average error in the CoM positions of simulated diffraction peaks on the virtual detector (*i.e.* detector CoM_*y*_, detector CoM_*z*_ and CoM_ω_ angle) with respect to signal-to-noise variation. For clarity, the CoM_*y*_ and CoM_ω_ curves are plotted with offsets of 0.5 µm and 0.1°, respectively. (*b*) The relative errors in the number of detector pixels per peak (*i.e.* the cumulative area of the observed intensity in pixel units) and in the integrated intensities of the harvested peaks with respect to signal-to-noise variation. The signal-to-noise level is expressed by the parameter β (see text).

Fig. 7 ▸(*a*) reveals a similar trend for the error on calculated peak positions as a function of intensity, starting to degrade below β = 0.4. Likewise, in Fig. 7 ▸(*b*) the integrated intensities and the average number of detector pixels per peak are seen to decrease in a logarithmic manner for β → 0. Note that the associated standard deviations of these metrics are also a strong function of signal-to-noise (S/N) level. At the extreme case of 95% intensity loss, the determined CoM position of peaks on the detector frame has an inaccuracy of up to ∼1 µm.

The results illustrated in Figs. 6 ▸ and 7 ▸ show that the proposed HR-3DXRD setup enables sub-pixel accuracy in the determination of diffraction spots. The average accuracy does not degrade until intensity levels are reached where many peaks are lost as their intensities are below a threshold. This behaviour extends to the full subgrain mapping example from the noisy data. Despite missing ∼85% of the available diffraction peaks, the pipeline has successfully determined 90% of the subgrains with 0.16 µm accuracy. These findings have two practical implications:

(i) The number of determined peaks shows a clear cut-off with respect to S/N. In actual experiments, this information can be exploited by performing a series of short ω-wedge scans with different exposure times, followed by peak harvesting. This exercise could provide an online measure for the quality of acquired data and thus help to find the best compromise between speed and data quality.

(ii) Due to its small pixels and large FoV, in experiments with low S/N conditions HR-3DXRD is still capable of producing full or partial 3D maps of subgrains without losing its high spatial and angular accuracy.

### 4.2. Demonstration of HR-3DXRD reconstruction of simulated microstructures

In order to examine the resolving capabilities of HR-3DXRD, virtual experiments were carried out with three different phantoms, one, Phantom *R*, with a random texture and two, Phantoms *A* and *B*, representing a grain in a deformation microstructure of a material with medium to high stacking-fault energy. In these simulations there is no noise and the alignment of the instrument is assumed to be perfect.

Phantom *R*, which has randomly orientated (sub)grains, mimics the undeformed microstructure of *e.g.* high-entropy alloys. It comprises 5000 subgrains with an average grain size of 1 µm. Here, the size of each grain is estimated through calculating the diameter of an ‘equivalent sphere’ that has the same volume. The indexing tolerances used are 0.018° in 2θ, 0.1° in η and 0.05° in ω. The resulting FoMs for the CoM map and the derived subgrain volumes are summarized in Table 1 ▸. It appears that all subgrains have been identified correctly, with position, orientation and volume errors that are so small that tessellations based on CoM maps for the original phantom and reconstructed CoM maps are, to all intents and purposes, identical. In passing, we note that these results are much superior to those of standard 3DXRD on the same number of grains, a testimony to the order of magnitude better spatial resolution of HR-3DXRD.

**Figure 8:**
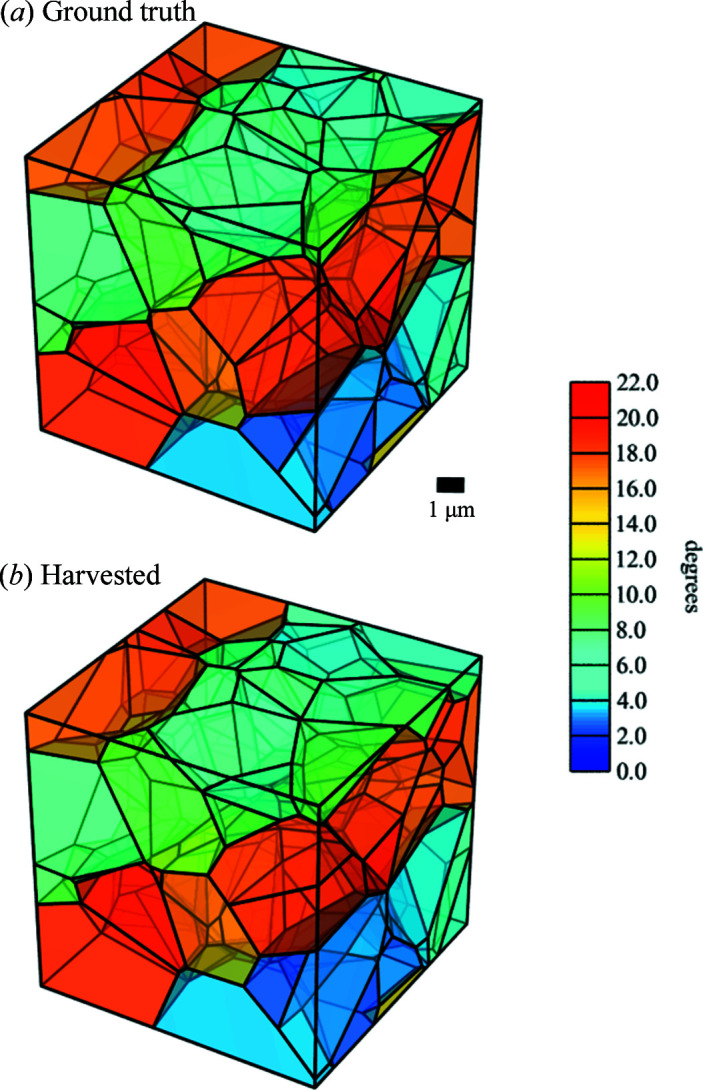
3D tessellated maps of Phantom *A*, (*a*) for the ground truth and (*b*) based on the determined grain parameters obtained from the analysis of the simulated diffraction images. Scale legends for length and misorientation are given. Subgrains are contained in a cube-shaped box with a side length of 10 µm. For clarity, the misorientations shown are calculated against an orientation reference that is 12° away from the original average orientation of the phantom.

Phantoms *A* and *B* are constructed using the multiscale tessellation tool of the *Neper* software (Quey, 2019 ▸; Quey *et al.*, 2018 ▸). Inspiration for these phantoms was taken from the characteristics of the microstructure of rolled metals of medium to high stacking-fault energy (Huang & Winther, 2007 ▸). More specifically, microstructural parameters for rolled aluminium at ε_*vM*_ = 0.3 have been employed (Liu *et al.*, 1998 ▸). The phantoms are generated by the *Neper* software using as input the size of the simulation box (*i.e.* the ‘volume of interest’), the plane and spacing of the GNBs, the mean cell size, the width of the cell size distribution, and the mean misorientation across GNBs and IDBs. The graphical result for Phantom *B* is illustrated in Fig. 3 ▸. The underlying list of the CoM positions and crystallographic orientations of individual subgrains is fed as input to *PolyXsim* to generate detector images. The same list is fed back into *Neper* to reconstruct the phantoms by tessellation without the enforcement of planar boundaries, yielding the microstructures in Figs. 8 ▸(*a*) and 9 ▸(*a*). The final tessellations based on the analysis of the harvested peaks in Figs. 8 ▸(*b*) and 9 ▸(*b*) are generated in the same way for visual comparison.

Phantom *A* is presented in Fig. 8 ▸(*a*). It consists of three GNBs and a total of 104 subgrains in a cube-shaped box with a side length of 10 µm. Phantom *B* [Figs. 3 ▸ and 9 ▸(*a*)] is a larger version of Phantom *A* consisting of six GNBs and 828 subgrains in a cube-shaped box with a side length of 20 µm. The plane normal of the GNBs aligns with the crystallographic (001) plane. The mean crystallographic misorientation in the two phantoms is 7° and the mean spacing between IDBs is 1.25 µm, which is about half the GNB spacing. As illustrated by the colours in Figs. 8 ▸(*a*) and 9 ▸(*a*), the misorientation between subgrains delineated by two GNBs is smaller than the misorientation across the GNBs. In addition, the misorientation between neighbouring GNBs alternates in sign.

For both phantoms, synthetic data sets were produced and analysed following the description in Section 3.2. The indexing tolerances used for Phantom *A* are 0.005° in 2θ, 0.025° in η and 0.05° in ω, and for Phantom *B* 0.013, 0.025 and 0.025°, respectively (both tighter than for the randomly orientated phantom).

Table 2 ▸ lists the results for Phantom *A*. The number of input peaks for indexing shows that the peak harvesting procedure has found 94% of the peaks theoretically available. The indexing successfully identified all 104 subgrains, with ∼0.1 µm average error in CoM position and less than 0.001° error in orientation. For the same data set, the largest CoM and orientation errors are 0.242 µm and 0.002°, respectively. Table 2 ▸ also shows that all assigned diffraction spots have found a match in the corresponding ground-truth subgrain’s peaks, with errors lower than the synthetic detector pixel size and ω rotation step. On average, the indexed subgrains had a purity of 88%, suggesting that there were no false assignments to cause inaccuracies in the 2θ and η angles. Furthermore, the volume of the indexed subgrains is determined with a relative absolute error of around 4%.

Table 3 ▸ lists the corresponding results for Phantom *B*. The substantially higher local density in the pole figure [see Fig. 3 ▸(*b*)] implies that the fraction of harvested peaks is reduced compared with Phantom *A*: 28% of peaks are missing. Furthermore, only 772 subgrains are found – and this with ∼60% purity. There are no false positives: all subgrains match a subgrain in the original phantom. The statistics for the 56 unidentified ground-truth subgrains did not vary from the statistics on the indexed subgrains in terms of subgrain size and CoM position. Moreover, the unidentified subgrains are found to be homogeneously distributed in different GNBs and they follow a similar distribution to the mean subgrain size of identified subgrains. Hence, we believe the origin of ‘lack of indexing’ is related to the statistics of how close adjacent reflections happen to be on the detector.

The identified subgrains still exhibit very small average CoM position errors (of the order of 0.1 µm) and orientation errors (less than 0.001°). The highest observed CoM and orientation errors are 0.367 µm and 0.002°, respectively. On the diffraction spot level, the errors in the 2θ and η angles show a similar behaviour to the Phantom *A* case, with calculated errors that are well below the synthetic detector pixel size and smaller than the ω rotation step. The volume of the indexed subgrains is determined with a relative error of 5%.

**Figure 9:**
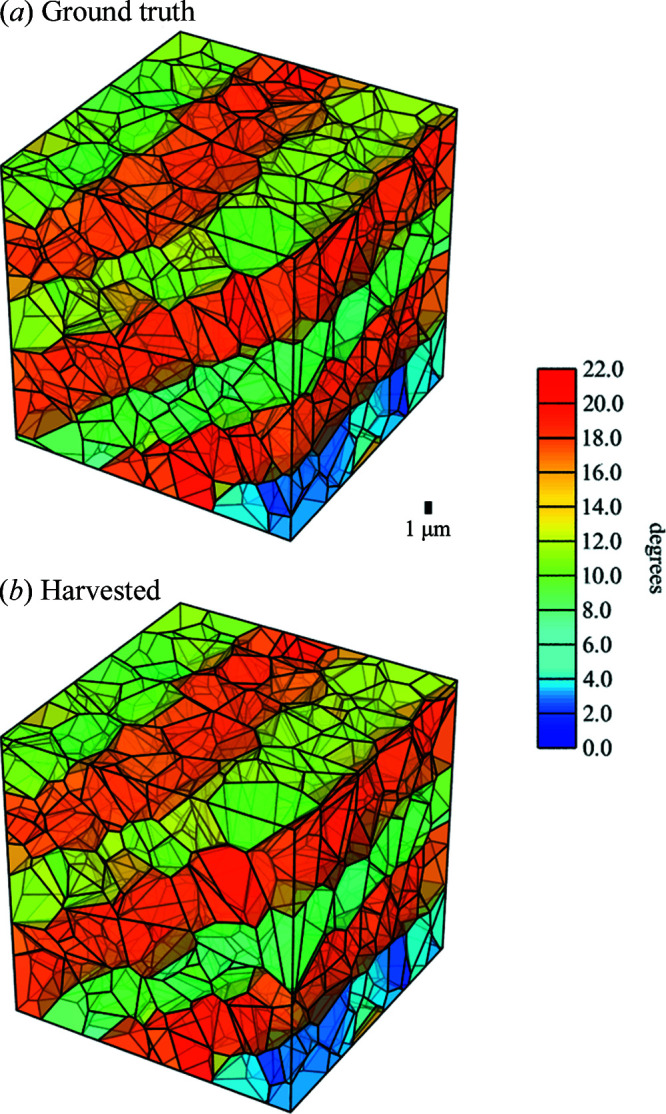
3D tessellated maps of Phantom *B*, (*a*) for the ground truth and (*b*) based on the determined grain parameters obtained from the analysis of the simulated diffraction images. Scale legends for length and misorientation are given. Subgrains are contained in a cube-shaped box with a side length of 20 µm. For clarity, the shown misorientations are calculated against an orientation reference that is 12° away from the original average orientation of the phantom.

Tessellated maps of the reconstructions of the two deformed phantoms were generated according to Section 3.3. The result for Phantom *A* is compared with a tesselated map of the initial input to *PolyXSim*, here termed the ground truth, in Fig. 8 ▸. The tessellation of the ground truth is successfully replicated by identifying all three GNBs and all their constituent IDBs, together with their corresponding misorientation to the seed orientation. Likewise, the tessellated 3D maps of Phantom *B* are given in Fig. 9 ▸. Although the harvested analysis in this case misses 7% of the ground-truth subgrains, all the GNBs present are still clearly visible. Moreover, except for the nearest-neighbour vicinity of the missing subgrains, the tessellated subgrain map is of high quality. Lyckegaard *et al.* (2011 ▸) showed that tessellations from a data set with 10% volume error would lead to acceptably low errors, *i.e.* 0.6 extra neighbours per subgrain and 0.6 missed neighbours per subgrain. Comparing with the current results, our analysis shows comparably lower intensity errors for both Phantoms *A* and *B*. Likewise, the average positional error for Phantom *B* is 3.5% of the average subgrain diameter. Hence, we expect that tessellations of both phantoms have a good definition of the subgrain neighbourhood with significantly lower errors.

## 5. Discussion

The hypothesis underlying this paper is that the *a priori* known structural properties of plastically deformed crystals can be exploited to enable 3D mapping of subgrains. This has been numerically tested and verified.

### 5.1. Detector requirements and other limitations

For the experimental setting simulated in Section 4, a 225 megapixel camera would be required. The current state of the art in high-resolution X-ray imaging is represented by a PCO (Germany) cooled camera with 26 megapixels, 2.5 µm pixel size and a 66 dB dynamic range (https://www.pco.de/scientific-cameras/pcoedge-26-clhs/), while standard imaging cameras tend to have just 4 megapixels. However, the state of the art in professional photography is represented by 151 megapixel CMOS cameras with 3.7 µm pixel size from the company PhaseOne (Denmark). We consider it likely that this technology will migrate to X-ray imaging, and this prospect is a primary motivation for establishing HR-3DXRD.

In practice, fewer pixels may be required. First of all, if spot overlap is not an issue, multigrain indexing can be performed on the basis of three or even two complete Debye–Scherrer rings, rather than the six used for the tests in Section 3. This reduces the demand on the number of pixels by a factor of 4 or more, but does also reduce the fidelity of the indexing.

Likewise, the angular spread of the diffraction spots ξ may not be governed by the diffraction limit, ξ ≃ λ/*d*. While it is possible to condense the incident beam with a divergence ξ ≤ λ/*d* (*e.g.* using compound refractive lenses), in some cases this divergence may be the governing contribution. Then the optimal sample-to-detector distance, *L*, will be shorter than *N*_F_ = 1 and given by ξ*L* = *d*, where *d* is the typical subgrain size. The reduced *L* will reduce the requirement in terms of number of pixels, but will also reduce the efficiency of HR-3DXRD.

**Figure 10:**
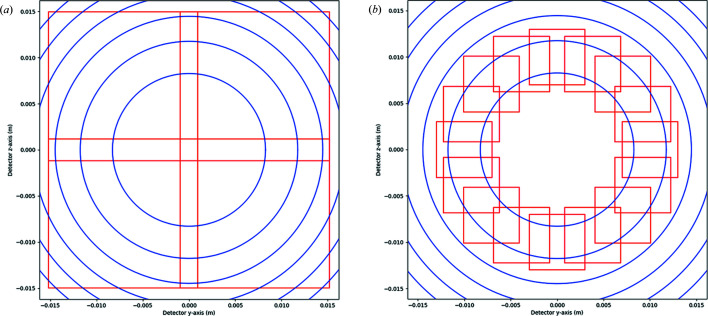
Two possible experimental realizations of HR-3DXRD using (*a*) a 30.25 megapixel detector and (*b*) a 4 megapixel detector. In both cases, the detector is moved perpendicular to the optical axis. The individual images then form regions of interest (red boxes) of a larger virtual compound image. The circles represent the first six Debye–Scherrer rings of α-Fe shown in the same configuration as employed in Section 3.

As a replacement for a monolithic detector, a compound detector image may be synthesized from images acquired by one or more smaller FoV 2D detectors that are offset with respect to the optical axis. Two such schemes are shown in Fig. 10 ▸ with reference to the experiment simulated in Section 3. In both situations, a single 2D detector is moved within a plane perpendicular to the incident beam, and images are acquired consecutively for different sub-regions of the virtual compound image. The four-panel solution shown on the left-hand side of Fig. 10 ▸ could reflect the realization of HR-3DXRD with an existing 30.25 megapixel camera, while the 24-panel solution shown on the right-hand side might show the realization with an existing 4 megapixel camera.

The high spatial accuracy of HR-3DXRD implies that the positions and tilts of the various panels must be calibrated with a corresponding accuracy. To facilitate this, an external calibration sample may not be sufficient. Hence, we propose that the FoV of the panel regions should overlap. Image registration can then be used to identify the same spots in the overlapping regions, and neighbouring panels can be aligned using cross-correlation methods. In a second refinement step the geometric parameters (position, tilt) for all panels may be optimized as part of the global fitting and refinement of all subgrains (*cf*. Fig. 4 ▸).

The first experimental HR-3DXRD data sets have recently been acquired by translating a 2000 × 2000 detector with 3 µm pixel size in the manner presented in Fig. 10 ▸(*b*). The details and results of this experimental implementation of HR-3DXRD are outside the scope of this paper and will be presented elsewhere.

Provided a suitable detector is available, the present work points to the prime limitation of HR-3DXRD being spot overlap. Sørensen *et al.* (2012 ▸) showed that an overlap ratio below 10% is acceptable for 3DXRD and we anticipate this limit will also be valid for HR-3DXRD. (Notably, the orientation variation within each subgrain is ≤1 mrad, and therefore the overlap can be minimized by taking small steps in ω.) The severity of this limitation will depend strongly on the material, grain size and degree of plastic deformation.

### 5.2. Application to deformed polycrystals

Phantoms *A* and *B* represent a single plastically deformed grain. When studying polycrystals, other grains than the one of interest will be simultaneously illuminated. However, most of these grains will rotate in and out of the illuminated volume during data acquisition. The relatively low number of harvested reflections from such grains implies that the reflections will be filtered out by the algorithm. Neighbouring grains in close proximity to the one of interest are an exception. However, there will be relatively few of these, provided there is no dominant texture, and therefore the overlap of orientation spreads will be low [Fig. 3 ▸(*b*)].

### 5.3. Comparison with existing methods

The resulting 3D orientation maps are similar to those obtained by combining EBSD (Wright & Adams, 1992 ▸; Krieger Lassen, 1998 ▸; Steinmetz & Zaefferer, 2010 ▸) with serial sectioning by means of fast-ion bombardment milling (Uchic *et al.*, 2006 ▸) or femtosecond laser-beam ablation (Echlin *et al.*, 2012 ▸; Lenthe *et al.*, 2015 ▸). HR-3DXRD exhibits a much improved orientation resolution, revealing the existence of ultra-low-angle boundaries, similar to studies previously done using gallium-enhanced microscopy (Hagstrøm *et al.*, 2003 ▸). On the other hand, the spatial resolution of EBSD is better and the prime motivation for using X-rays is their non-destructive sampling.

Scanning 3DXRD is conceptually a more general-purpose method that has less difficulty with handling strong local textures. The data analysis route is simpler. The prime motivation for HR-3DXRD is the speed of data acquisition. The advantage will be strongly dependent on the specifications of the beamline, the material, the grain size and the degree of deformation. To give an estimate, for the science case discussed in Section 3, a 20 × 20 × 20 µm deformed grain and a resulting map with 200 nm resolution, at the ESRF EBS it requires one day of acquisition for scanning 3DXRD and 10 min for HR-3DXRD with a potential 100 megapixel detector.

### 5.4. Outlook

EBSD and 3DXRD methods and associated software have matured over decades. Developing the full potential of HR-3DXRD is likely to include additional concepts. Experimental verification of feasibility on recovered specimens is seen as the next step. Following this, a generalization to include the determination of the elastic strain tensor for each subgrain is highly relevant, as are studies of materials with a non-cubic crystal structure.

It is likely that HR-3DXRD may benefit from adapting existing 3DXRD concepts. An example (*e.g.* for strain determination) may be the use of two or three sample-to-detector distances and ‘tracking’, a concept originally developed for the indexing of grains (Lauridsen *et al.*, 2001 ▸).

Another example is coupling to Mode I operation, as defined by Poulsen (2020 ▸). Here, the temporal limitation is overcome by first generating a 3D map of all subgrains and then probing the dynamics of a selected subset of these by acquiring images only within a small ω range. The work of Ahl *et al.* (2020 ▸) illustrates the latter aspect. Here, the structural properties of 500 individual and deeply embedded subgrains in a deformed Al polycrystal were followed during recovery with a time resolution of 1 s.

The data analysis pipeline comprises indexing and refinement software that were originally developed for samples of low texture. It is likely that the highly textured case considered here could benefit from an indexing algorithm that relies not only on orientation information but also on position information (*e.g.* from Friedel pairs; Ludwig *et al.*, 2009 ▸; Bernier *et al.*, 2011 ▸) and/or relative intensities (Poulsen, 2004 ▸). Likewise, one may exploit the hierarchical nature of data (grains, bands, subgrains) in both direct and reciprocal space.

Finally, we underline the point that the application area of HR-3DXRD extends beyond deformation microstructures to other materials with grains or domains in the 1 µm range, *e.g.* high-entropy alloys, additive manufacturing parts *etc*.

## 6. Conclusions

Characterization of a deformed microstructure by means of orientation mapping is a cornerstone of metallurgy. In this paper, we have presented a numerical demonstration of a method for non-destructive 3D mapping of deformation microstructures. Using polycrystal diffraction simulations, we have explored the possible limitations and the applicability of the new modality. Our findings can be summarized as the following:

(i) For near-perfect crystals with sizes of ∼1 µm, the spatial and angular resolution of a 3DXRD-type experiment can be simultaneously enhanced by placing a high-resolution imaging detector at a distance corresponding to a Fresnel number of approximately 1 and making CoM maps. However, to be efficient this modality requires the use of a 2D detector with tens or hundreds of megapixels. The prospect of using such detectors has been discussed.

(ii) Uniquely for a full-field imaging method, HR-3DXRD is capable of revealing and mapping the individual subgrains in metal microstructures. Their orientations are determined with ∼0.0005° resolution.

(iii) Analysis of HR-3DXRD data may be pursued with currently available tools for Mode II far-field 3DXRD, but requires a careful selection of tolerances.

(iv) HR-3DXRD is quite robust towards misalignments and S/N issues, while spot overlap is likely to be the main limitation. If most reflections are harvested, the subgrains are correctly indexed. The dominant error in the resulting 3D maps is missing subgrains, which may be identified by studying the local geometric properties of the tessellations. By inspection of regions with no missing subgrains, we see that neighbours are identified with high fidelity.

(v) HR-3DXRD is still relevant if a fraction of subgrains are missing. The main features of a deformed microstructure, namely the bands of subgrains in between GNBs, can still be identified.

## Figures and Tables

**Table 1:** Quality of the reconstructed subgrain map for Phantom *R*, comprising 5000 randomly oriented subgrains

	No. of subgrains	No. of peaks	Purity (%)	2θ error (°)	η error (°)	ω error (°)	Position error (µm)	Orientation error (°)	Volume error (%)
Ground truth	5000	1 404 501	–	–	–	–	–	–	–
Results	5000	1 291 942	87.4 ± 4.1	0 ± 8 × 10^−5^	0 ± 5 × 10^−5^	0 ± 1 × 10^−3^	0.09 ± 0.03	1 × 10^−4^ ± 9 × 10^−5^	0.02 ± 1.40

**Table 2:** Quality of the reconstructed subgrain map for deformed Phantom *A*

	No. of subgrains	No. of peaks	Purity (%)	2θ error (°)	η error (°)	ω error (°)	Position error (µm)	Orientation error (°)	Volume error (%)
Ground truth	104	29 684	–	–	–	–	–	–	–
Results	104	27 835	88.3 ± 0.1	0 ± 3 × 10^−5^	0 ± 9 × 10^−5^	0 ± 3 × 10^−4^	0.09 ± 0.04	5 × 10^−4^ ± 6 × 10^−4^	−1.45 ± 3.63

**Table 3:** Quality of the reconstructed subgrain map for deformed Phantom *B*

	No. of subgrains	No. of peaks	Purity (%)	2θ error (°)	η error (°)	ω error (°)	Position error (µm)	Orientation error (°)	Volume error (%)
Ground truth	828	235 898	–	–	–	–	–	–	–
Results	772	169 652	59.6 ± 15.6	0 ± 2 × 10^−5^	0 ± 5 × 10^−5^	0 ± 3 × 10^−4^	0.09 ± 0.04	5 × 10^−4^ ± 6 × 10^−4^	5.12 ± 4.03

## Footnotes

1 As described by Ehrenfest (1927 ▸), the CoMs of waves propagate in straight lines irrespective of the nature of the wave or emitter.

2 The choice of pixel size was based on an experimental configuration in which a high-resolution detector with a native pixel size of 5.86 µm was utilized with an optical objective of 2× magnification.
